# Pharmacokinetic and Pharmacodynamic Responses to Clopidogrel: Evidences and Perspectives

**DOI:** 10.3390/ijerph14030301

**Published:** 2017-03-14

**Authors:** Yan-Jiao Zhang, Mu-Peng Li, Jie Tang, Xiao-Ping Chen

**Affiliations:** 1 Department of Clinical Pharmacology, Xiangya Hospital, Central South University, Changsha 410008, China; zhangyj287112687@163.com (Y.-J.Z.); elskesunny@163.com (M.-P.L.); jietang@csu.edu.cn (J.T.); 2 Institute of Clinical Pharmacology, Hunan Key Laboratory of Pharmacogenetics, Central South University, Changsha 410078, China

**Keywords:** clopidogrel, pharmacogenomics, genetic polymorphisms, epigenetics, non-genetic factors

## Abstract

Clopidogrel has significantly reduced the incidence of recurrent atherothrombotic events in patients with acute coronary syndrome (ACS) and in those undergoing percutaneous coronary intervention (PCI). However, recurrence events still remain, which may be partly due to inadequate platelet inhibition by standard clopidogrel therapy. Genetic polymorphisms involved in clopidogrel’s absorption, metabolism, and the P2Y12 receptor may interfere with its antiplatelet activity. Recent evidence indicated that epigenetic modification may also affect clopidogrel response. In addition, non-genetic factors such as demographics, disease complications, and drug-drug interactions can impair the antiplatelet effect of clopidogrel. The identification of factors contributing to the variation in clopidogrel response is needed to improve platelet inhibition and to reduce risk for cardiovascular events. This review encompasses the most recent updates on factors influencing pharmacokinetic and pharmacodynamic responses to clopidogrel.

## 1. Introduction

Dual antiplatelet therapy with aspirin and P2Y12 inhibitors is crucial for patients with acute coronary syndrome (ACS) and post percutaneous coronary intervention (PCI) to prevent future thrombotic events [1]. Clopidogrel, an oral irreversible P2Y12 receptor antagonist, is widely used in clinical practice in comparison to other P2Y12 antagonists such as ticagrelor or prasugrel. After intestinal absorption, approximately 85% of the clopidogrel prodrug is hydrolyzed by esterase into an inactive form, leaving only 15% of clopidogrel transforming to the active metabolite by the hepatic cytochrome P450 (CYP450) system, of which *CYP2C19* is a crucial enzyme. However, vast studies have shown broad inter-individual variability in the antiplatelet effect of clopidogrel. Impaired platelet responsiveness to clopidogrel may result in increased risk of cardiovascular events [2,3]. Numerous studies have demonstrated the association between *CYP2C19* polymorphisms and the antiplatelet effect of clopidogrel. Also, other factors including epigenetics, demographics, concurrent diseases, and drug-drug interactions may contribute to the poor response. Our review attempts to demonstrate the comprehensive components affecting pharmacodynamics and pharmacokinetics that can explain the mechanisms underlying clopidogrel response variabilities.

## 2. Genetic Polymorphisms in Drug Disposition and Drug Targets

**Figure 1:**
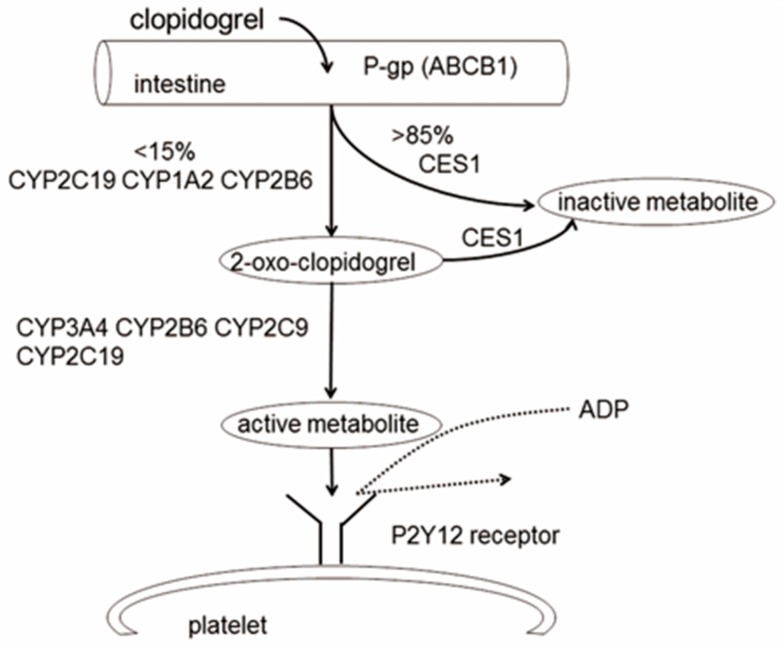
The metabolic pathway of clopidogrel and its targeted receptor. Intestinal absorption of the prodrug clopidogrel is limited by P-glycoprotein (P-gp). After absorption, the clopidogrel (inactive) is oxidized to 2-oxo clopidogrel (still inactive) by CYP450 enzymes. The 2-oxo clopidogrel is then transformed into active metabolites that will bind to P2Y12 receptor on platelet surfaces.

Polymorphisms in genes responsible for the drug efflux *(ABCB1)*, metabolic activation or inactivation (*CES1*, *CYP2C19*, *CYP3A4/5*), and biological activity (*P2RY12*, *PEAR1*) of clopidogrel may account for a part of the interindividual variability in clopidogrel response (Figure 1, Table 1).

### 2.1. ABCB1 Polymorphisms

Clopidogrel functions only when absorbed by the intestine after oral administration. Evidence has shown that clopidogrel absorption is limited by the intestinal efflux transporter P-glycoprotein (P-gp) encoded by *ABCB1* gene. Taubert et al. firstly demonstrated that variable intestinal clopidogrel absorption was influenced by the *ABCB1* C3435T polymorphism in 60 patients with coronary artery disease [4]. Then Simon et al. found that carriers of the *ABCB1* 3435 TT genotype had a higher rate of cardiovascular events than CC homozygotes in patients with acute myocardial infarction (AMI) [5]. Subsequent clinical studies and meta-analysis verified the association between *ABCB1* 3435TT genotype and impaired platelet response as well as the higher risk of major adverse cardiovascular events [6,7]. Several trials have shown that *ABCB1* 3435T was associated with lower levels of plasma clopidogrel and its active metabolite [8,9,10]. However, there are also inconsistent reports on the association of *ABCB1* polymorphism and clopidogrel response. For example, a recent meta-analysis including six studies with 10,153 subjects failed to show an association between the *ABCB1* C3435T polymorphism and the risk of overall recurrent ischemic events, stent thrombosis, or bleeding in clopidogrel treated patients [11], which was further confirmed by Jaitner et al. in patients undergoing PCI [12].

### 2.2. CES1 Polymorphisms

Carboxylesterase (CES) is the most predominant hydrolytic enzyme in the human body. CES catalyzes the hydrolysis of numerous ester- and amide-containing endogenous compounds, toxins, and medications to their respective free acids. The vast majority of absorbed clopidogrel is shunted by CES1 to inactive carboxylic metabolites [41]. Therefore, genetic variations affecting CES1 expression or its activity are supposed to be important determinants of clopidogrel response. *CES1* has two isotypes, *CES1A1* (often called *CES1*) and *CES1P1*. Previous studies have identified several single nucleotide polymorphisms (SNPs) in the coding region of *CES1*, including rs71647871 (G143E), rs71647872 (D260fs), and the intronic variant rs8192950 [13,42]. There is a study showing that the rs8192950 polymorphism is associated with a decreased risk of clinical events in clopidogrel treated patients with extracranial or intracranial stenosis [13]. The frameshift mutation rs71647872 is extremely rare. The G143E results in the non-conservative amino acid substitution of Glycine 143 to Glutamic acid, which decreases CES1 catalytic activity [42]. Lewis et al. found that carriers of the *CES1* 143E allele have higher levels of the clopidogrel active metabolite and better clopidogrel response than the 143G allele (wild-type) in healthy people [14]. Meanwhile, in patients with coronary heart disease treated with clopidogrel, the lower ADP-induced platelet aggregation and lower risk of cardiovascular events were found in 143E allele carriers. Tarkiainen et al. also reported in healthy volunteers that *CES1* 143E carriers have a larger AUC of clopidogrel and the active metabolite and lower P2Y12-mediated platelet aggregation [15]. In addition, *CES1P1* rs3785161 was found to be associated with attenuated antiplatelet effect of clopidogrel in 162 coronary heart disease patients [16].

### 2.3. PON1 Polymorphisms

Paraoxonase-1 (PON1) is an esterase synthesized in the liver and associated with HDL (high density lipoprotein)-cholesterol. A previous study has shown that PON1 is a crucial enzyme for clopidogrel biotransformation to the active metabolite through hydrolytic cleavage of the c-thiobutyrolactone ring of 2-oxo-clopidogrel [17]. The *PON1* Q192R (rs662) variant was initially reported to activate clopidogrel more efficiently [17]. However, the following investigations did not replicate the results of Bouman et al. [18]. Mega et al. reported that the Q192R genetic variant was not associated with the pharmacologic or clinical response to clopidogrel, and their results of the meta-analysis including 13 studies also demonstrated no statistically significant association between the 192Q variant and major adverse cardiac events (MACE) during clopidogrel therapy [19]. Moreover, the work by Dansette et al. showed that the second step enzymatic conversion mainly depends on the P450 pathway from 2-oxo-clopidogrel to 4b cis, but also depends on PON1 to minor metabolite 4b “endo”, whose antiplatelet activity was not determined yet [43]. These results suggest that the role of PON1 in clopidogrel resistance may not be important.

### 2.4. CYP2C19 Polymorphisms

The CYP2C19 isoenzyme is involved in the two-step reaction of clopidogrel activation. Hulot et al. firstly found the association between the *CYP2C19* loss-of-function (LOF) allele (*2) and a marked decrease in platelet responsiveness to clopidogrel in young healthy male volunteers in 2006 [20]. Several following studies have demonstrated the association between *CYP2C19* genetic variants (*2, *3, and *17) and the risk of adverse cardiovascular outcomes in clopidogrel-treated patients [21,22,23,24,25]. A genome-wide association study (GWAS) demonstrated that the CYP2C19*2 variant was associated with poor clopidogrel response (*p* = 4.3 × 10^−11^) in healthy people and increased ischemic events (*p* = 0.02) during a 1-year follow-up in patients [23]. Wei et al. confirmed that CYP2C19*2 was associated with higher rate of clopidogrel resistance and ischemic events [21]. Although Bhatt et al. failed to observe the association in patients with stable angina [22], two large meta-analyses have demonstrated the significant association between *CYP2C19* LOF and recurrent cardiovascular events in different ethnic patients [44,45]. Meanwhile, the *CYP2C19* LOF had a significant reduction of AUC_0–t_, the concentration of clopidogrel, or active metabolite [24,26,46]. In March 2010, the U.S. Food and Drug Administration (FDA) even announced a boxed warning on clopidogrel, stating that *CYP2C19* LOF which harbors two reduced function alleles (*2 and *3) reduces CYP2C19 catalytic activity and attenuates the efficacy of clopidogrel. After that, the American College of Cardiology Foundation and the American Heart Association published a consensus document addressing this FDA warning [47]. However, *CYP2C19* LOF allele carriage accounts for only 5% to 12% of the overall variability of the clopidogrel response [23].

A *CYP2C19* gain-of-function (GOF) allele (*17) in the 5-flanking region of the gene is observed to be associated with increased *CYP2C19* transcription [48]. This GOF allele confers a rapid metabolism of CYP2C19 substrates, which may lead to a higher concentration of clopidogrel active metabolite, an enhanced antiplatelet activity, and an increased risk of bleeding events during clopidogrel therapy [27]. Hamsze et al. also confirmed that the CYP2C19*17 polymorphism was associated with decreased on-treatment platelet reactivity and increased risk of major bleedings [27,28]. In the 2013 updated Clinical Pharmacogenetics Implementation Consortium Guidelines for *CYP2C19* Genotype and Clopidogrel Therapy, *CYP2C19* genotype-guided clopidogrel therapy was recommended to ACS patients managed with PCI [49].

### 2.5. CYP3A4/5 Polymorphisms

CYP3A consists of the 3A4 and 3A5 isoenzymes and is responsible for the conversion of 2-oxo clopidogrel into active clopidogrel metabolites. Therefore, reduced CYP3A4/5 activity is supposed to decrease clopidogrel response. Between the two isoenzymes, CYP3A4 is the dominant form and CYP3A5 acts as a so-called “backup system” in situations where drugs may act as inhibitors of CYP3A4 [50].

CYP3A4*1G has been reported to be associated with decreased CYP3A4 expression [29]. However, Danielak et al. reported that influence of CYP3A4*1G was not found on either the pharmacokinetics or pharmacodynamics of clopidogrel [51].

The CYP3A5*3 allele, a functional SNP located in intron 3, results in a premature truncated protein associated with null enzymatic activity, and CYP3A5*3/*3 homozygotes lack CYP3A5 protein expression and activity in the liver [52]. The influence of CYP3A5*3 polymorphism on clopidogrel response may be dependent on *CYP2C19* genetic status and CYP3A4 inhibitors. Patients with the CYP3A5*3/*3 genotype exhibited higher platelet reactivity compared to carriers of the CYP3A5*1 allele in *CYP2C19* poor metabolizers [30]. This may help to explain that when reduced CYP2C19 activity and CYP3A4 substrates or inhibitors occur, the CYP3A5 backup system for CYP3A4 would play a role. Nakkam N et al. also reported that the impact of CYP3A5*3 on clopidogrel response is pronounced in subjects carrying *CYP2C19* LOF [31]. In patients treated with amlodipine, a CYP3A4 inhibitor, *CYP3A5* non-expressers (*3/*3 homozygotes) showed higher on-treatment platelet reactivity [32].

### 2.6. P2RY12 Polymorphisms

Activation of P2Y12 receptor leads to sustained platelet aggregation via the phosphoinositide 3-kinase (PI3K) pathway to activate glycoprotein IIb/IIIa. Fontana et al. identified three SNPs and one nt insertion in the *P2RY*12 (i-C139T, i-T744C, G52T, i-ins801A), and two haplotypes called H1 and H2 [33] were inferred from the four polymorphisms; carriers of the H2 haplotype exhibited enhanced platelet activity. In another study, Rudez et al. identified haplotype F was associated with higher on-clopidogrel platelet reactivity [34]. However, a subsequent study failed to find an association between *P2RY12* polymorphism (T774C) and clopidogrel responsiveness in 597 ACS patients [35]. Therefore, more studies are necessary to corroborate the relation between *P2RY12* genetic polymorphisms and clopidogrel response.

### 2.7. PEAR1 Polymorphisms

Platelet Endothelial Aggregation Receptor-1 (PEAR1), a platelet transmembrane protein, is associated with platelet aggregation and endothelial function. *PEAR1* polymorphisms have been shown to influence platelet reactivity after antiplatelet therapy [36]. *PEAR1* rs41273215 and rs57731889 were independent predictors of high on-treatment platelet reactivity and low on-treatment platelet reactivity, respectively, in Chinese coronary heart disease after PCI [37]. Other SNPs, including rs2768759 and rs11264579, were also reported to increase platelet activity [38,39]. Lewis et al. also evaluated the impact of *PEAR1* rs12041331 polymorphism on platelet aggregation and clinical outcomes in three studies. They found that carriers of the rs12041331 A allele were more likely to experience cardiovascular events or die compared to those of GG homozygotes [40]. However, this observation suggested the effect of rs12041331 on post-aspirin platelet aggregation is mediated through collagen receptor pathways and not ADP-dependent pathways. Therefore, further studies are necessary to define the precise role of *PEAR1* polymorphism in antiplatelet therapy.

## 3. Epigenetics Influencing Clopidogrel Response

Though pharmacogenetics studies have found several SNPs associated with clopidogrel response, the effect of most polymorphisms on the individual variation of platelet activity has not been fully confirmed except for *CYP2C19* LOF. Recent studies have indicated that the epigenetic modification of genes involved in drug disposition or effects can also affect the drug response. Epigenetic modification can affect gene expression and chromatin structure without altering the nucleotide sequence, and is influenced by physiological and pathological conditions and environmental factors as well. Interest in the epigenetic study of clopidogrel response has also increased in recent years. Most of these studies regarding clopidogrel are focused on microRNA and DNA methylation.

### 3.1. MicroRNAs

MicroRNAs (miRNAs) are single stranded, short, and small noncoding RNA of ~22 nucleotides in length [53]. MiRNAs can reduce mRNA expression by binding to the target mRNA directly and interfering with protein translation [54]. Numerous studies have explored the link between specific mRNAs and miRNAs to platelet reactivity and activation [55,56,57]. For example, Kondkar et al. have demonstrated that miR-96 regulates the expression of platelet vesicle-associated microtubule protein 8 (VAMP8), a critical component of platelet granule exocytosis [56]. Girardot et al. also indicated that miR-28 regulates the expression of the thrombopoietin receptor directly [57].

In 377 miRNAs observed in human platelets, miR-223 was the most differentially expressed miRNA in platelet-rich plasma compared with platelet-poor plasma and serum [58]. Circulating platelet miRNAs are also supposed to act as indicators for tailoring antiplatelet therapies. MiR-223 is in the highest level among platelet miRNAs and could suppress *P2RY12* mRNA level in HEK293 cells [59]. Decreased miR-223 expression in platelet and plasma predicted high on-treatment platelet reactivity in clopidogrel treated patients [60,61], which indicated that the miR-223 level might serve as a potential biomarker to predict clopidogrel response. MiR-26a was found to participate in the regulation of platelet reactivity by clopidogrel via regulating the expression of vasodilator-stimulated phosphoprotein [62].

### 3.2. DNA Methylation

DNA methylation is specially observed in the context of the cytosine phosphate guanine (CpG) dinucleotide and is mostly studied in promoter regions or gene bodies and represses gene transcription [63].

*ABCB1* promoter methylation was reported to suppress *ABCB1* mRNA and protein expression in tumor cells [64,65,66]. Hypomethylation of *ABCB1* promoter was associated with poor response to clopidogrel in Chinese ischemic stroke patients with CYP2C19*1/*1 genotype [67]. ABCC3, another member of the ABC family, was associated with the efflux of clopidogrel and its antiplatelet activity [68,69]. However, *ABCC3* promoter methylation and down-regulation of *ABCC3* mRNA had no significant association with clopidogrel response [70]. Different detection methods of platelet activity and different subjects result in different conclusions. Therefore, further studies are needed to corroborate the conclusion. Hypomethylation of *P2RY12* promoter was associated with clopidogrel resistance in coronary artery disease (CAD) patients with smoking, albumin <35 g/L, and alcohol abuse [71]. A recent epigenome-wide study revealed that lower methylation of cg03548645 within *TRAF3* body was associated with increased platelet aggregation and vascular recurrence in ischemic stroke patients treated with clopidogrel [72]. They hypothesized that higher *TRAF3* expression due to decreased methylation may lead to an increase in the CD40 signal pathway interfered platelet-platelet interactions [73,74].

## 4. Non-Genetic Factors Influencing Clopidogrel Response

Variation in platelet inhibitory effects of clopidogrel has been shown to be associated with the genetic factors, including polymorphisms and epigenetics. However, those data of genetic variations are insufficient to explain the varieties in clopidogrel response. Non-genetic factors, such as demographic characteristics, concurrent diseases, and drug interactions, are also observed to have an influence on the antiplatelet effect of clopidogrel.

### 4.1. Demographic Characteristics

Age and BMI were significantly associated with clopidogrel response [75,76]. Older age was independently associated with a higher rate of high residual on-treatment platelet reactivity with both clopidogrel and ticagrelor in 494 patients on dual antiplatelet therapy [77]. Additionally, obesity (BMI ≥ 30 kg/m^2^) was independently associated with higher residual platelet reactivity in clopidogrel-treated patients [78].

Several trials have demonstrated that smokers show better clopidogrel responsiveness than nonsmokers, which is called the smokers’ paradox. Gurbel et al. assessed the effect of smoking on clopidogrel and prasugrel therapy in patients with CAD. Lower clopidogrel active metabolite exposure and decreased antiplatelet effects of clopidogrel were observed in nonsmokers compared to smokers, but the same phenomenon was not observed for prasugrel, another P2Y12 antagonist [79]. Park et al. found that better clopidogrel response could be reversed after discontinuation of smoking, which further confirmed the causal relationship between smoking and clopidogrel [80]. An explanation for the smokers’ paradox is CYP1A2 and CYP2B6 induction by cigarette smoking, which results in greater formation of the clopidogrel active metabolite [79,81]. Also, *CYP1A2* rs762551 polymorphism showed influence on clopidogrel response only in smokers [81]. However, a recent study observed a significant inverse correlation between the VerifyNow P2Y12 reaction unit and hemoglobin levels in current smokers receiving clopidogrel therapy, and the difference in the platelet reaction unit (PRU) between nonsmokers and current smokers disappeared after adjusting for the effect of hemoglobin on PRU [82]. The exact mechanisms for these observations require further study. However, the results of Ferreiro et al. and Zhang et al. are contrary to these findings [83,84]. Their studies showed that cigarette smoking is an independent risk factor for adverse ischemic outcomes with a single antiplatelet agent or in non-cardioembolic ischemic stroke patients. Moreover, in smokers there was a trend of lower composite vascular events in clopidogrel-treated patients as compared with the aspirin-treated patients, whereas no such trend was observed in never-smokers. It is necessary to confirm under which circumstances the effect of the smokers’ paradox on clopidogrel will be established.

### 4.2. Complications

#### 4.2.1. Diabetes Mellitus

Patients with diabetes mellitus (DM) account for the most proportion of the population worldwide. The prevalence of cardiovascular diseases (CVDs) rises to as high as 55% in DM patients, and CVDs account for 65% of deaths in DM patients [85]. Diabetic patients have a poor antiplatelet effect of clopidogrel treatment compared to non-diabetic patients [86,87]. The mechanism of this phenomenon has been explored by some investigators but is inconsistent. Insulin can regulate platelet activity through the insulin receptor substrate-1 (IRS-1) on platelets. Type 2 DM is characterized by reduced insulin sensitivity which may lead to increased platelet activity and decreased antiplatelet activity. *IRS-1* rs956115 and rs13431554 polymorphisms were associated with high platelet activity and increased risk of adverse events in type 2 DM CAD patients [88,89]. Moreover, Angiolillo et al. explored the mechanism of clopidogrel response variability in 60 diabetic and non-diabetic patients treated with aspirin and clopidogrel [90]. The maximal plasma concentration of the clopidogrel active metabolite and the area under the concentration-time curve was lower in the diabetic group. In addition, significantly higher platelet activity was observed in vitro incubation of clopidogrel active metabolites with platelets from DM patients as compared to those from non-DM patients. These results suggested that poor clopidogrel response in DM is mainly due to the decreased concentration of the active metabolite but also partly attributed to the upregulation of the P2Y12 signaling pathway [90]. When the antiplatelet effect of clopidogrel is influenced by DM, switching to other potent antiplatelet drugs may be considered. Results from the TRITON-TIMI 38 trial have also revealed that prasugrel tends to provide a greater reduction in ischemic events than clopidogrel in diabetic patients compared with non-diabetic patients with ACS [91]. In the PLATO trial, a consistent benefit with ticagrelor over clopidogrel, including reduced mortality, was observed irrespective of diabetic status [92]. The cilostazol plus clopidogrel therapy achieved greater platelet inhibition compared with clopidogrel alone in T2DM and *CYP2C19* LOF variants [93]. Therefore, the novel and more potent P2Y12 receptor inhibitors (prasugrel and ticagrelor) or cilostazol may be a better strategy to overcome clopidogrel poor response in patients with DM.

#### 4.2.2. Chronic Kidney Disease

Chronic kidney disease (CKD, creatinine clearance <60 mL/min) is a common comorbidity of patients with atherosclerotic vascular disease. CKD was associated with increased risk of recurrent cardiovascular and bleeding events in PCI-treated patients [94,95]. Siddiqi et al. also reported a higher risk of death, myocardial infarction, and bleeding in patients with CKD [96]. It is suggested that prolonging clopidogrel beyond the standard guidelines of 12 months after PCI may help to reduce this long-term increased risk in patients with CKD [96]. However, other researchers drew inconsistent conclusions. Mangiacapra et al. found no association between residual platelet reactivity and CKD when the VerifyNow P2Y12 assay was used, but some association between CKD and ischemic and bleeding events [97]. Baber et al. found that the association between residual platelet reaction and CKD was attenuated after multivariable adjustment, which implied that confounding risk factors, rather than renal dysfunction itself, account for the high platelet reactivity (HPR) [98]. Moreover, the HPR could result in increased risk of ischemic and bleeding events irrespective of CKD status. A large meta-analysis also failed to show the definite association between antiplatelet therapy and CKD [99], suggesting that this association needs more studies to be proven.

### 4.3. Drug Interactions

Numerous studies are focused on the drug-drug interactions between the proton pump inhibitor, calcium channel blocker, statin, morphine, or caffeine and clopidogrel.

#### 4.3.1. Proton Pump Inhibitor

Both clopidogrel and aspirin can show the symptoms of gastrointestinal bleeding. Patients with clopidogrel treatment are usually recommended for proton pump inhibitors (PPIs) to prevent gastrointestinal complications such as ulceration and bleeding. Omeprazole and esomeprazole, two moderate CYP2C19 inhibitors, have been reported to decrease clopidogrel efficacy and increase risk of adverse clinical outcomes [100,101]. The weak CYP2C19 inhibitor pantoprazole has less effect on the clopidogrel response [101]. In 2010, the FDA and the European Medicines Agency issued a warning on concomitant administration of clopidogrel and PPIs, especially for omeprazole and esomeprazole. Clinically, more patients are recommended for pantoprazole treatment. However, some observational studies also showed discordant findings for the concomitant use of PPIs. Chiara et al. suggested that PPIs as a class were associated with worse clinical outcomes in observational studies of patients, with unstable angina/non-ST-segment elevation myocardial infarction patients receiving clopidogrel and aspirin therapy, but omeprazole showed no difference in ischemic outcomes as compared with the placebo [102]. The COGENT trial demonstrated that omeprazole therapy did not lead to an increased risk of cardiovascular events but significantly attenuate the gastrointestinal risk [103]. These controversial conclusions reveal that further prospective research is still needed to determine the clinical significance of clopidogrel-PPI interactions.

#### 4.3.2. Calcium Channel Blocker

As some calcium channel blockers (CCBs) can inhibit CYP3A4, a key enzyme in the conversion of clopidogrel, the concomitant use of CCBs might compete with clopidogrel for the CYP3A4 enzyme, which can also result in an impaired response to clopidogrel. Several studies have reported the impacts of CCBs on the platelet reactivity and clinical outcomes of clopidogrel [104]. CYP3A4*1G allele decreased the CYP3A4 enzyme activity, and was associated with an increased vulnerability to the effects of CCBs on clopidogrel response variation [105]. Park et al. indicated that taking a 600 mg loading dose of clopidogrel may reduce this impact [105]. Meanwhile, some CCBs also show strong inhibitory effects on P-gp and lead to decreased intestinal efflux of clopidogrel, thereby increase plasma concentration of clopidogrel. Therefore, the coadministration of P-gp inhibiting CCBs (such as verapamil, nifedipine, diltiazem, and barnidipine) can counteract the reduced effect of CCBs on the metabolic activation of clopidogrel, while non-P-gp inhibiting CCBs such as amlodipine still diminish the efficacy of clopidogrel [106].

#### 4.3.3. Statin

Statins, known as HMG-CoA reductase inhibitors, are widely used to treat hypercholesteremia. As CCBs, it is possible that CYP3A4-metabolized statins (atorvastatin, simvastatin) also influence clopidogrel response. Lau et al. firstly reported that atorvastatin other than pravastatin reduced the antiplatelet effect of clopidogrel by controlling CYP3A4 activity in a dose-dependent manner [107]. Horst et al. also reported that pre-treatment with atorvastatin and simvastatin reduced the inhibitory effects of clopidogrel significantly in patients with CAD [108]. Additionally, the platelet inhibitory effect of clopidogrel was enhanced by replacement of atorvastatin with a non-CYP3A4-metabolized statin in patients with high platelet reactivity [109]. However, these results did not conform to several subsequent studies [110,111,112], which revealed no significant difference between atorvastatin and other statins affecting the clinical efficacy in ACS patients receiving clopidogrel therapy. Saw et al. also did not observe adverse interaction between clopidogrel and statins after long-term use [112]. Therefore, clinical significance of this putative drug-drug interaction is still controversial. These conflicting observations may be due to differences in the assessment assay of platelet activity, use of different doses or classes of statin, and patient selection.

#### 4.3.4. Morphine

Morphine is sometimes recommended for the treatment of pain from myocardial infarction. Hobl et al. performed a randomized, double-blind, placebo-controlled, cross-over trial in 24 healthy volunteers receiving a 600 mg loading dose of clopidogrel simultaneously with 5 mg morphine or placebo. They found that morphine delayed the T_max_ of clopidogrel and decreased the AUC_0−n_ of clopidogrel active metabolite by 34%. In addition, the time to maximal inhibition in platelet aggregation was two-fold longer than the placebo group. As morphine can delay gastric emptying, it is possible that morphine might delay clopidogrel absorption, decrease peak plasma levels, and decrease its antiplatelet activity [113,114]. Interestingly, this phenomenon was also seen in both prasugrel and ticagrelor, suggesting that morphine and oral antiplatelet interaction is non-drug specific and is most likely explained by morphine’s gastrointestinal side effects [115]. The randomized, double-blind, placebo-controlled IMPRESSION trial showed that morphine lowered the total exposure to ticagrelor and its active metabolite by 36% and 37%, respectively, with a concomitant delay in maximal plasma concentration of ticagrelor. Moreover, a higher platelet reactivity was found in the group that received morphine [116]. Therefore, the use of morphine should be careful during the use of oral antiplatelet medications.

#### 4.3.5. Caffeine

Tea and coffee that contain caffeine are the most widely consumed beverages in daily life. A few investigations have exhibited the influence of caffeine intake on the cardiovascular system [117]. Clopidogrel inhibits the platelet P2Y12 receptor, leading to increased intracellular cyclic adenosine monophosphate (cAMP) level. It is observed that caffeine can also increase cAMP accumulation and alter the aggregation of platelets by upregulating the adenosine A2A receptor [118]. Acute caffeine administration was reported to increase the platelet inhibition effect of clopidogrel in healthy subjects and CAD patients [119]. The extent of influence of caffeine on clopidogrel responsiveness requires further examination.

## 5. Conclusions

In recent years, the adverse cardiovascular events resulting from clopidogrel resistance have raised increasing concerns. Therefore, it is meaningful to determine the specific factors contributing to the variability in clopidogrel clinical treatment. However, the mechanisms underlying poor clopidogrel response have not yet been fully elucidated. In this review, we systematically summarized numerous factors affecting the pharmacodynamics and pharmacokinetics of clopidogrel. Most of these factors (e.g., CYP2C19*2/*3 and DM) may contribute to the low active metabolite of clopidogrel and/or high on-treatment platelet reactivity, which predict increased risk of thrombosis, ischemic events, and cardiovascular death. While other factors (CYP2C19*17 and smoking) may have the opposite effect, increasing clopidogrel active metabolite exposure and improving clopidogrel response. The impact of some factors (e.g., PON1 and CKD) cannot be fully determined on the response to clopidogrel treatment. In short, it is difficult to determine only one factor or only a few factors at a time that result in clopidogrel resistance. Increasing drug dosage or switching to alternative drugs such as ticagrelor or prasugrel is preferred for those patients with impaired antiplatelet effects of clopidogrel.

## Figures and Tables

**Table 1:** Genetic polymorphisms observed to be associated with clopidogrel response.

Polymorphisms	Samples	Influence on Pharmacokinetics and Pharmacodynamics	Influence on Clinical Outcome	References
*ABCB1* C3435T	60 CAD	Lower exposure to clop-AM	NA	[4]
	2208 AMI	NA	Increase in cardiovascular risk	[5]
	2188 PCI-treated	Higher on-treatment platelet reactivity	Increase in cardiovascular risk	[6]
	NA	No negative effect on platelet reactivity	Increase in cardiovascular risk	[7]
	401 ACS	Lower exposure to clop-AM and CLP	NA	[8]
		Higher on-treatment platelet reactivity		
	123 AMI	Lower exposure to CLP and 2-oxo- CLP	NA	[9]
	42 PCI-treated	Lower exposure to CLP	NA	[10]
	10153 subjects	NA	Inconclusive	[11]
	1524 PCI-treated	inconclusive	NA	[12]
*CES1* rs8192950	377 ischemic stroke	NA	Decrease in cardiovascular risk	[13]
G143E	566 healthy volunteers	Higher exposure to clop-AM	NA	[14]
	350 CAD	Lower on-treatment platelet reactivity		
	1109 healthy volunteers	Higher exposure to clop-AM	NA	[15]
		Lower on-treatment platelet reactivity		
*CES1P1 rs3785161*	162 CAD	Higher on-treatment platelet reactivity	NA	[16]
*PON1 Q192R*	Many groups	Higher exposure to clop-AM	Decrease in cardiovascular risk	[17]
		Lower on-treatment platelet reactivity		
	482 CAD	Inconclusive	NA	[18]
	275 healthy volunteers	Inconclusive	Inconclusive	[19]
	2922 ACS			
CYP2C19*2*3	28 healthy volunteers	Higher on-treatment platelet reactivity	NA	[20]
	110 ACS	Higher on-treatment platelet reactivity	Increase in cardiovascular risk	[21]
	4819 atherothrombosis	NA	Decrease in bleeding risk but not increase in cardiovascular	[22]
	429 healthy volunteers	Higher on-treatment platelet reactivity	Increase in cardiovascular risk	[23]
	227 PCI-treated			
	162 healthy volunteers	Lower exposure to clop-AM		[24]
		Higher on-treatment platelet reactivity		
	1477 ACS		Increase in cardiovascular risk	
	259 MI	NA	Increase in cardiovascular risk	[25]
	366 CAD	Lower exposure to CLP	NA	[26]
*17	1524 PCI-treated	Lower on-treatment platelet reactivity	Increase in bleeding risk but not in cardiovascular	[27]
	820 CVD	Lower on-treatment platelet reactivity	Increase in bleeding risk	[28]
CYP3A4*1G	82 PCI-treated	Inconclusive	NA	[29]
CYP3A5*3	101 angina	Higher on-treatment platelet reactivity	NA	[30]
	35 healthy volunteers	Higher on-treatment platelet reactivity	NA	[31]
	1258 PCI-treated	Higher on-treatment platelet reactivity	Increase in cardiovascular risk	[32]
*P2RY12* H2	98 healthy volunteers	Higher on-treatment platelet reactivity	NA	[33]
A-F	1031 CAD	Higher on-treatment platelet reactivity	NA	[34]
T774C	597 ACS	Inconclusive	NA	[35]
*PEAR1* rs12041331	104 healthy volunteers	Platelet aggregation	NA	[36]
rs56260937				
rs41273215	204 CHD	Higher on-treatment platelet reactivity	NA	[37]
rs57731889		Lower on-treatment platelet reactivity		
rs2768759	1486 healthy volunteers	Higher on-treatment platelet reactivity	NA	[38]
rs11264579	500 healthy volunteers	Higher on-treatment platelet reactivity	NA	[39]
rs12041331	565 healthy volunteers	Higher on-treatment platelet reactivity	Increase in cardiovascular risk	[40]
	227 PCI-treated			
	1000 CAD			

ACS: acute coronary syndrome; MI: myocardial infarction; AMI: acute myocardial infarction; CHD: coronary artery disease; clop-AM: clopidogrel active metabolite; CLP: clopidogrel; NA: not available.
